# Bioconversion of fructus sophorae into 5,7,8,4’-tetrahydroxyis oflavone with *Aspergillus aculeatus*

**DOI:** 10.1371/journal.pone.0211613

**Published:** 2019-03-06

**Authors:** Yuqian Zhang, Yunchen Zhao, Yaoying Lu, Qiming Cao, Wenzhong Chen, Yuru Chen

**Affiliations:** 1 College of Food and pharmaceutical engineering, Nanjing Normal University, Nanjing, P. R. China; 2 School of Biotechnology and Chemical Engineering, Taizhou College, Nanjing Normal University, Taizhou, P. R. China; 3 School of Agriculture and Biotechnology, Hexi University, Gansu Zhangye, P. R. China; 4 School of Natural Sciences, Griffith University, Nathan, Queensland, Australia; 5 School of Resources and Environment, University of Electronic Science and Technology of China, Chengdu, P. R. China; 6 Nanjing Luye Sike Pharmaceutical Co., Ltd, Nanjing, P. R. China; Tallinn University of Technology, ESTONIA

## Abstract

A fungus identified as Aspergillus aculeatus was used to biotransform genistein and glycosides to polyhydroxylated isoflavones. The strain was identified on the basis of colony morphology features and ITS rDNA sequence analysis. Phylogenetic tree was constructed to determine its taxonomic status. Genistein and glycosides were transformed by Aspergillus aculeatus to 5,7,8,4’- tetrahydroxyisoflavone. The chemical structure of the product was identified by high performance liquid chromatography(HPLC), liquid chromatography-mass spectrometry(LC/MS), Infrared spectroscopy (IR) and NMR spectrometer methods. The ITS rDNA sequence of the strain had 100% similarity with Aspergillus. Furthermore, it was ultimately identified as Aspergillus aculeatus. The metabolite of genistein and glycosides was identified as 5,7,8,4’-tetrahydroxyisoflavone. 120 mg 5,7,8,4’-tetrahydroxyisoflavone was made from 20 g fructus sophorae, which was bioconverted unconditionally by Aspergillus aculeatus for 96 h, and the purity was 96%. On the basis of the findings, Aspergillus aculeatus was a novel strain with specific ability to convert genistein and glycosides into 5,7,8,4’-tetrahydroxyisoflavone which had potential applications.

## Introduction

Isoflavones are a class of organic compounds and bio-molecules related to the flavonoids. They are thought of by some as useful in treating cancer. They are also very strong antioxidants. Genistin, genistein, rutin, quercetin and kaempferol are the main effective components of flavones and isofavonoids [1–2]. In recent years, the metabolic process of isoflavones and flavones has drawn more attention. Isoflavones such as genistein and daidzein are found in a number of plants including lupin, fava beans, soybeans, Kudzu, and psoralea being the primary food source[3–4], also in the medicinal plants[5–7], coffee[8] and Maackia amurensis cell cultures[9]. Fructus sophorae, Chinese name as “Huaijiao”, is widely used to treat heart diseases in China. It is effective against neoplasms, inflammation and hyperlipidemia. Besides, it can be used for curing senile osteoporosis, reducing uric acid concentration[10–11]. Studies showed that fructus sophorae contains flavonoids, isoflavonoids, alkaloids, terpenoids, amino acid, saccharide and phospholipids et al.

Genistein is an isoflavone that is described as an angiogenesis inhibitor and a phytoestrogen. Genistein can be metabolized by rats or human liver microsomes to hydroxylated metabolites of 5,6,7,4’-tetrahydroxyisoflavone, 5,7,8,4’-tetrahydroxyisoflavone, 5,7,3’,4’-tetrahydroxyisoflavone, 2,5,7,4’-tetrahydroxyisoflavone, 5,6,7,3’,4’-pentahydroxyisoflavone and 5,6,8,3’,4’-pentahydroxyisoflavone[12–13]. In this components, 5,7,8,4’-tetrahydroxyisoflavone, which is irreversible inhibitor of mushroom tyrosinase [14–15], is more effective for chromatodermatosis curing, food fresh keeping and pesticides making in agriculture et al. Through bioconversion to more active products, the antiproliferative activities of flavonoids were enhanced by CYP1 enzymes[16], and more and more tyrosinase inhibitors have been widely used in clinic[12]. Studies has to pay more attention on how to improve the yield of 5,7,8,4’-tetrahydroxyisoflavone and purity of the production. Studies showed that soybean isoflavones could be metabolized by fungi during soybean fermentation. Conversion of flavonoid glycosides to flavonols, quercetin and kaempferol, occurred in silkworm thorn leaves due to fermentation of lactobacillus[17]. Studies also showed sophoricoside from Fructus sophorae was metabolized to geistein by co-immobilized *Aspergillus niger* and Yeast[18]. And the metabolites of daidzein and genistein by *Aspergillus* strains were identified as 8-hydroxydaidzein and 5,7,8,4’-tetrahydroxyisoflavone respectively[19]. *Aspergillus* is one of oldest named genera of fungi. Aspergillus strains were used in traditional manufacturing of fermented foods are safe since those microbes have been eaten by people over a long time [20]. The fungal has distinctive nutritional strategy and the process of degradation is the mean of obtaining nutrients. *Aspergillus* represents a huge potential for finding new enzymes that could be used to convert plant biomass for their energy policy. In this study, we screened fungal strains which could improve highly the yield of isoflavonols from fructus sophorae on the basis of colony morphology features and ITS rDNA sequence analysis. Phylogenetic tree was constructed to determine its taxonomic status. Fermentation of fructus sophorae and the yield of 5,7,8,4’-tetrahydroxyisoflavone was also studied by using the strain fermentation in this paper.

## Experimental

### General methods

Morphology graph of the fungi was collected with Zeiss Axio Imager A1 microscope (Zeiss, Jena, Germany). IR spectra were obtained from a Nexus 670 spectrometer with scanning range of 4000–400 cm^-1^ (Nexus, Nicolet, USA). The NMR spectra were recorded on a Bruker AV-400 spectrometer (400MHz for ^1^H and ^13^C; Bruker, Faellanden, Switzerland) in DMSO-d6. HPLC was carried out on Agilent LC 1100 with an VWD detector (Agilent Technologies, Santa Clara, CA, USA). Semi-preparation HPLC was performed on Agilent 1200 with an VWD detector (Agilent Technologies, Santa Clara, CA, USA). LC-MS was conducted on an Agilent 6460 HPLC, coupled to negative electrospray ionization (ESI) tandem mass spectrometry (MS/MS) method. Mass spectra in the negative ion mode was operated under the following conditions: fragmenter voltage of 5 eV, voltage of 3500 V, nebulizer pressure of 45 psi, capillary temperature of 300°C, *m/z* range from 50 to 1000.

### Chemicals

Genistin (purity>99%) was purchased from Zelang Chemical Company of Nanjing. Dimethyl sulfoxide (DMSO) and other reagents were of the highest purity commercially available, or of HPLC grade. The fresh soil and fructus sophorae powder (80 mesh) was collected from Nanjing, Jiangsu Province, China.

### Culture medium

Activation medium was potato dextrose agar (PDA medium); Conversion medium contained 10% fructus sophorae extraction, 0.05 g MgSO_4_, 0.5 g NaNO_3_, 0.001 g FeSO_4_, 0.1 g KH_2_PO_4_, 1.8 g agar. Fructus sophorae powder (20 g) was extracted with 200 ml distilled water boiling for 30 minute. Fermentation medium was made of 10% fructus sophorae extraction.

### Isolation and identification of fungus

Preliminary identification of strain, includes colonial morphology, microscopic examination and a biotransformation test. The strains were isolated from the soil of campus Nanjing normal university Nanjing, Jiangsu Province of China. All the strains were cultured on solid medium at 30 °C for 3 days. Then DNA of strain was extracted by SK1201-UNIQ-10 after 16 h incubation. The ITS rDNA was amplified by using the universal primers of ITS1(TCCGTAGGTGAACCTGCGG) and ITS4(TCCTCCGCTTATTGATATGC). And amplification was carried out in 20μl buffer, which contained 1.0 μl template, 0.8 μl 5p primer down, 1.60 μl 2.5 mmol/L dNTP, and 0.2 μl Ex Taq. PCR amplification order was 5 min initial predegeneration at 95 °C, then 30 s degeneration at 95 °C, then 30s annealing at 58 °C, and final 30 s extension at 72 °C, total of 35 cycles. PCR extension time was 10 min at 72 °C. The sequencing was performed via the sequencing service at Shanghai Meiji Inc. Then ITS rDNA sequences were analyzed using the BLASTN tool of the National Center for Biotechnology Information(NCBI) to identify the strain with sequence similarity. Phylogenetic tree was constructed by the neighbor-joining method using the MEGA 6 program.

### Instrumental analysis of isolated metabolites

After 70 h—96 h of incubation, culture compound was centrifuged at 8000 rpm for 10 min. The supernatant (150 ml) was extracted with ethyl acetate(450 ml). The solute fraction was concentrated under vacuum to dryness. Then the fraction was re-dissolved by acetonitrile (10 ml).

Quantitative analysis of fructus sophorae mixture concentration and the fraction was carried out by HPLC of Agilent 1100 HPLC system. Sample analysis was carried out on C18 column (250 mm×4.6 mm I.D., 5 μm) at a column temperature of 30 °C. Water solution of 0.07% (v/v) phosphoric acid (A), and acetonitrile/methyl alcohol (Ratio of 12:30) (B) was the gradient elution. Isocraticed at early 12 min with 32% B then 8 min 54% B at a flow rate of 1.0mL/min. Injection volume was 20 μL and UV detection was at 260 nm.

The ethyl acetate fraction was then purified by HPLC using a 250×10 mm i.d., ODS2 spherisorb semipreparative C18 reversed-phase column. The gradient elution using water(A), acetonitrile/methyl alcohol (12:30)(B) consisted of an isocratic elution for 18 min with 45% B at a flow rate of 7 ml/min and 2 ml of sample injected with UV detection at 260 nm. The sample was collected at the maximum peak, and then water was removed by freeze drying method.

### Bioconversion experiments

Spores of *Aspergillus aculeatus* were added to fermentation medium and incubated at 30 °C, 160 rpm for 96h. Sample was collected at every 12 h, Then the samples were filtered by 0.45 μm filter membrane for structure detection based on HPLC/LC, MS and IR.

## Results and discussion

### Identification of the strain

**Fig 1:**
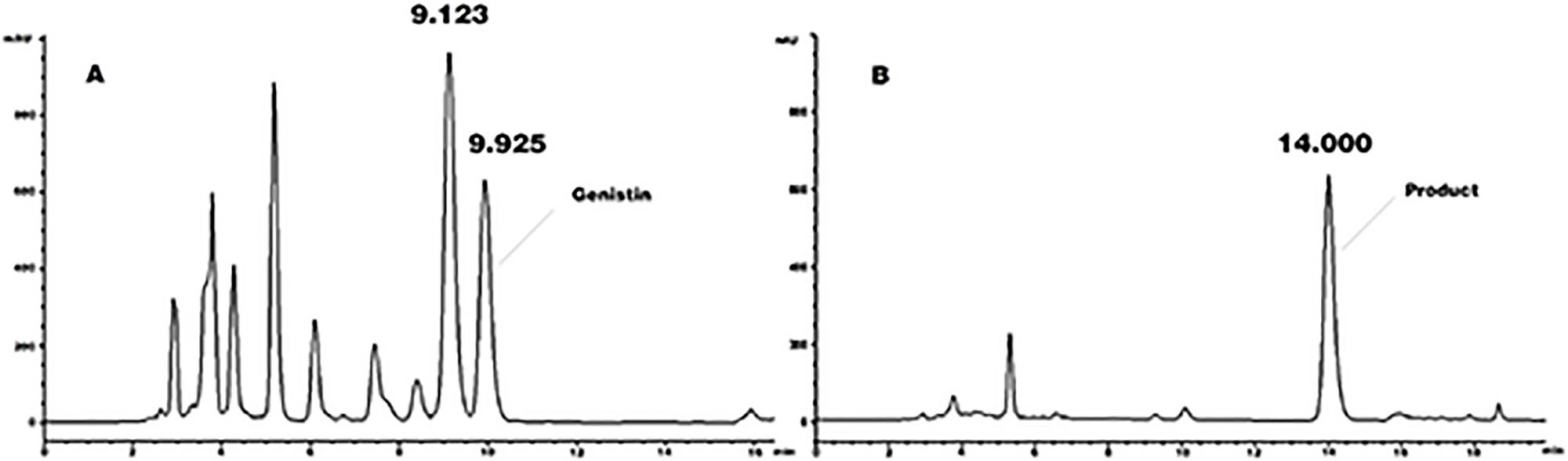
HPLC chromatograms of transformation mixture by Aspergillus aculeatus. 10% fructus sophorae extraction was used as substrate (A). Sample was collected at 96h(D).

Ten fungal strains were isolated from the soil. Then the transformable effects of the genistin of the ten strains were tested by inoculating the strains into the fermentation medium. The cultivations were analyzed by HPLC. After several days, the peak of genistin decreased only in the chromatograms of cultivations metabolized by *Aspergillus aculeatus* of all tested strains. Another peak(t_R_ = 14 min) appeared in the chromatograms of cultivations (Fig 1). These results indicated that only *Aspergillus aculeatus* metabolized genistin among the tested strains.

**Fig 2:**
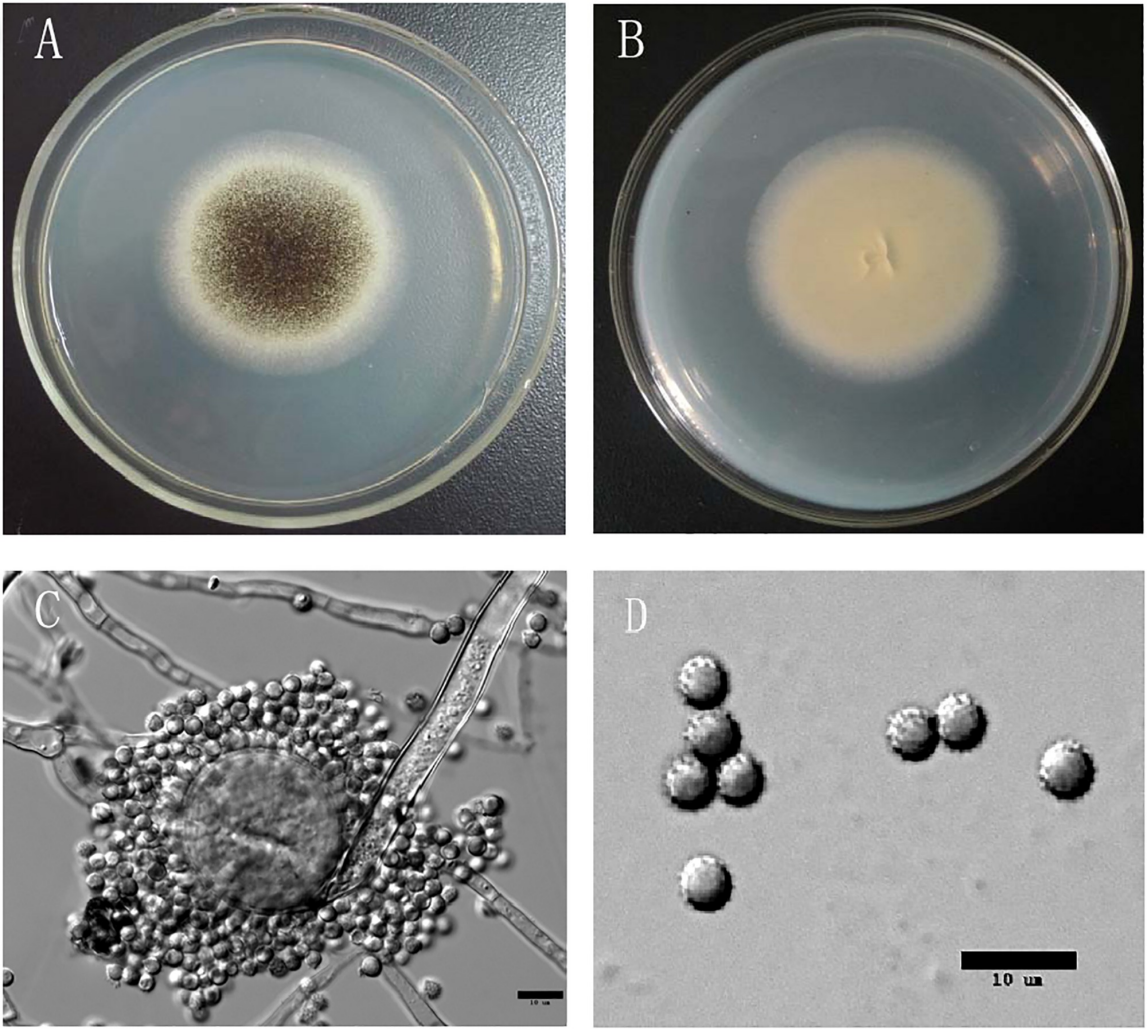
The morphology of Aspergillus aculeatus:Colonial morphology(A, B), microscopic examination(C, D).

Then the strain was identified by studying its morphology. After culturing for 3 days, the colour of *Aspergillus aculeatus* colony became black (Fig 2A). The color of the edge of colony was white, while the back was buff (Fig 2B). As shown under microscopy, the sporangium was spherical of 7–10 μm in diameter (Fig 2C). The spore was spherical with a diameter of 3.5–5 μm, which became brown and spiky on maturity (Fig 2D).

**Fig 3:**
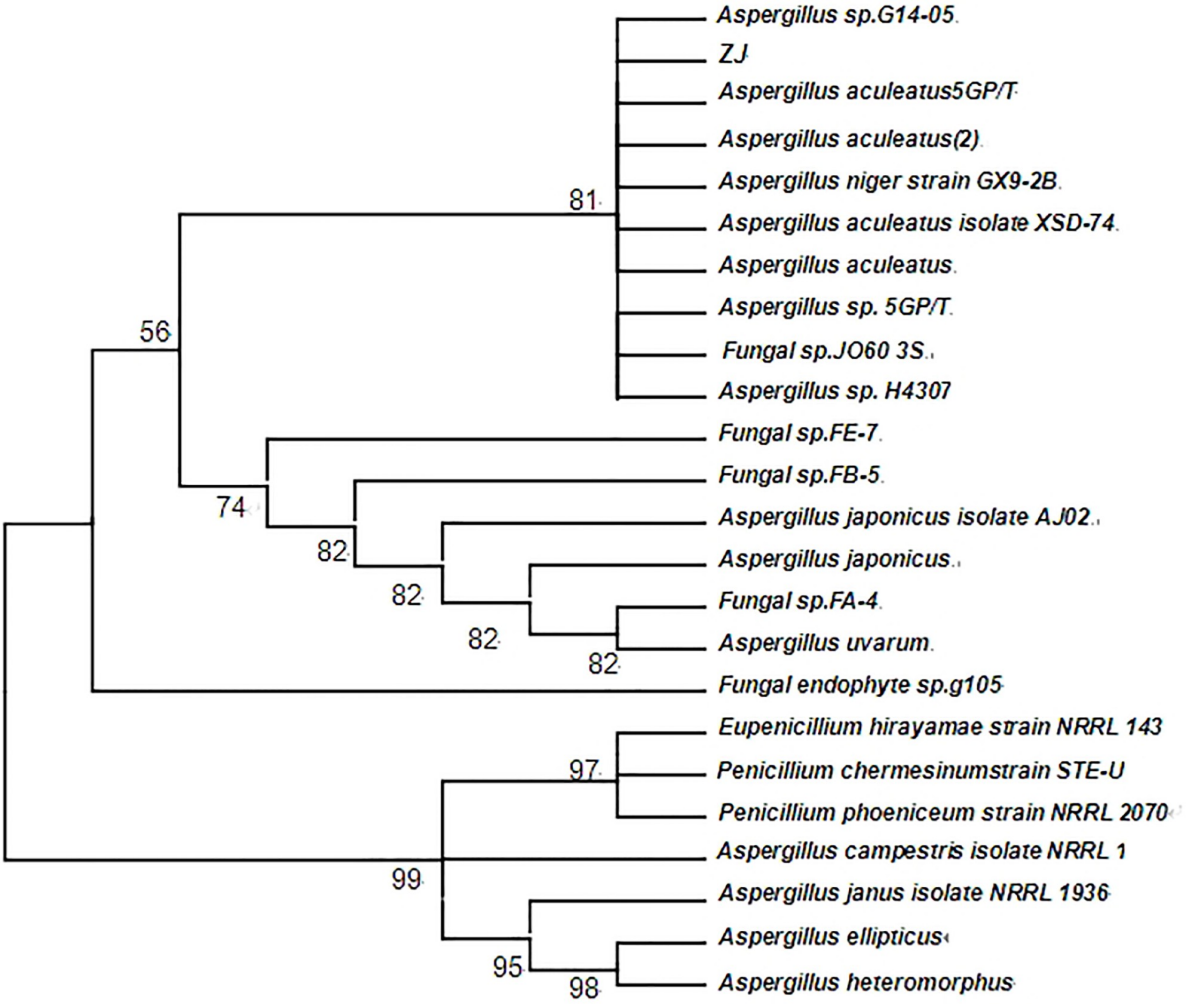
Phylogenetic tree of strain *Aspergillus aculeatus*.

The strain was identified on the basis of colony morphology features and ITS rDNA sequence analysis. ITS (Internal Transcribed Spacer) is a moderately conserved region. Its conservation is basically consistent within species, and the differences between species are obvious. This feature makes ITS suitable for molecular identification of fungal species. The ITS rDNA gene was 100% similar to *Aspergillus* whose template length was 1027 bases by comparation analysis with Blast and Gene Bank. Phylogenetic tree was constructed to determine its taxonomic status (Fig 3). Therefore, the strain was identified as *Aspergillus aculeatus*.

### Identification of metabolite

**Fig 4:**
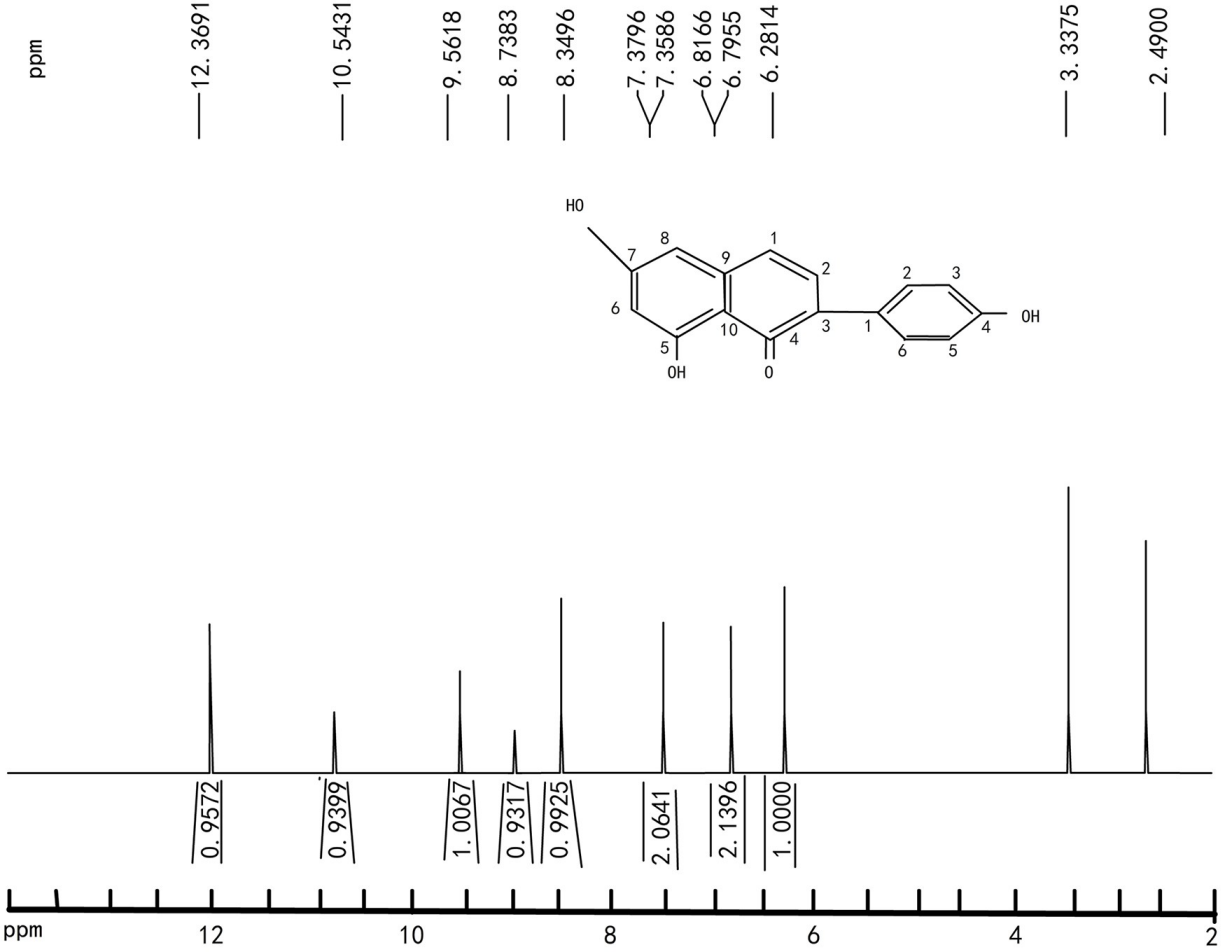
^1^H-NMR (400 MHz, DMSO-d6) spectrum.

The molecular formula of 5, 7, 8, 4’-tetrahydroxyisoflavone was determined by mass and NMR spectrometry. The ESI of 5, 7, 8, 4’-tetrahydroxyisoflavone showed pseudomolecular ion peaks at *m/z* 309.10 [M+Na^+^]^+^, 285.20 [M-H]^-^, which suggested the molecular formula of C_15_H_10_O_6_. The physicochemical properties of 5, 7, 8, 4’-tetrahydroxyisoflavone are given next:

^1^H-NMR(DMSO-d6): δ6.25(1H, S, H-6), 6.79(2H, d, H-3’, 5’), 7.35(2H, d, H-2’, 6’), 8.33(1H, S, H-2), 8.72(1H, S, OH-6), 9.54(1H, S, OH-4’), 10.52(1H, S, OH-7), 12.34(1H, S, OH-5)(Fig 4). ^13^C-NMR(DMSO-d6):δ181.019(C-4), 157.801(C-4’), 154.339(C-2), 153.948(C-5),153.683(C-7),146.343(C-9),130.672(C-2’,6’),125.353(C-8),122.250(C-1’), 121.852(C-3), 115.484(C-3’,5’), 104.543(C-10), 99.148(C-6).

As shown in Fig 4, the position of H-3 ', 5 ’and H- 2', 6' were determined to be in the B ring by the observation of doublet (δ6.79 ppm and δ7.35 ppm). And similar appears of H-2’and H-6’. H-2’ did not appear a sharp singlet at about δ6.30, Moreover, H-2’ was not precisely bind to the βcarbonyl group through carbon to oxygen. Above all, we concluded that the substance did not belong to flavonoids. It would further move to δ8.50 ~ 8.70 when used DMSO-d6 as a solvent. The chemical shift of 8.33 ppm corresponded to H-2, which confirmed that the substance should be isoflavones.

**Fig 5:**
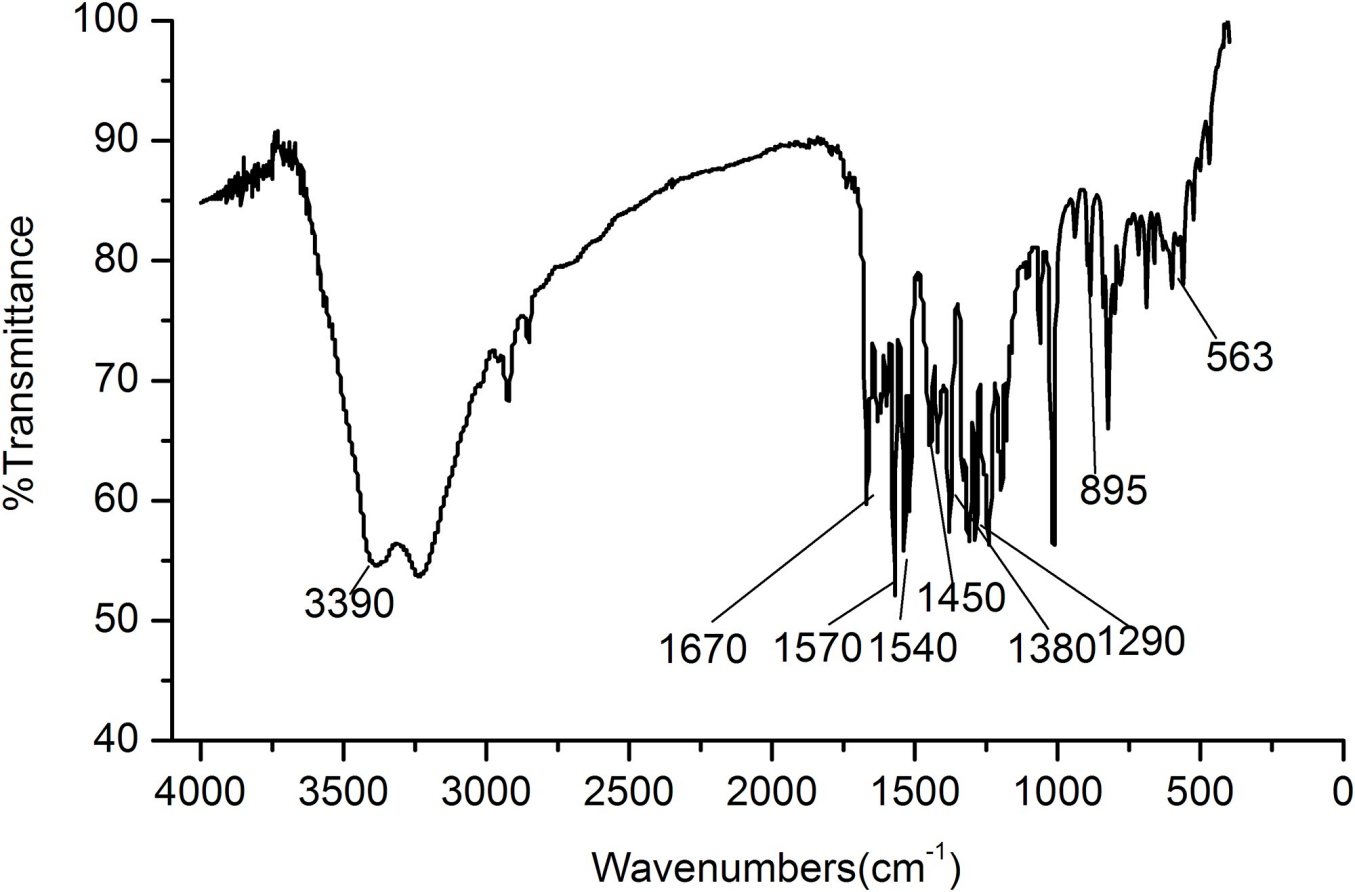
IR spectrum of 5, 7, 8, 4’-tetrahydroxyisoflavone.

The IR spectrum (KBr flaking, cm^-1^) (Fig 5) indicated the presence of hydroxyl (3390, 1380 and 1290cm^-1^), carbonyl(1670cm^-1^) and benzene ring(1540 and 1450cm^-1^). The bands between 665cm^-1^and 900cm^-1^ indicated the strongly interacting CH-stretching and bending vibrational modes.

### Fermentation of fructus sophorae

**Fig 6:**
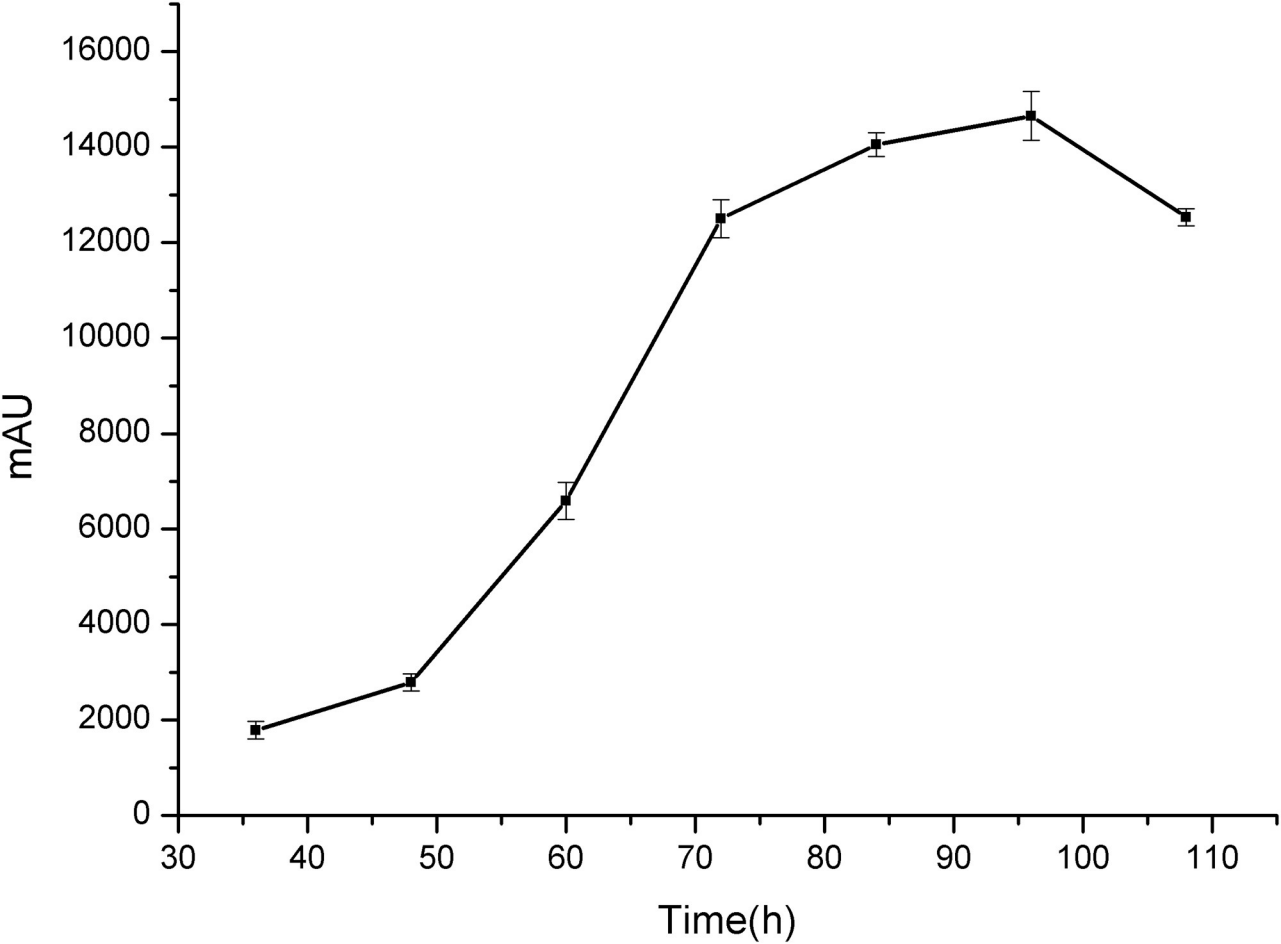
Courses of concentration of 5,7,8,4’-tetrahydroxyisoflavone in transformation.

*Aspergillus aculeatus* isolated from *Azadirachta indica* is an endophyte which had efficient antibacterial activity and broad spectrum. In this study, fructus sophorae was fermented by *Aspergillus aculeatus* for 120 h, sampling for each 12 h and only one component of 5, 7, 8, 4’-tetrahydroxyisoflavone was detected. This is not coined with the other research which showed that four compounds was detected in the fermentation [20]. After 96 h of fermentation, the concentration of 5, 7, 8, 4’-tetrahydroxyisoflavone reached to the maximum peak area then decreased with the fermentation process (Fig 6).

**Fig 7:**
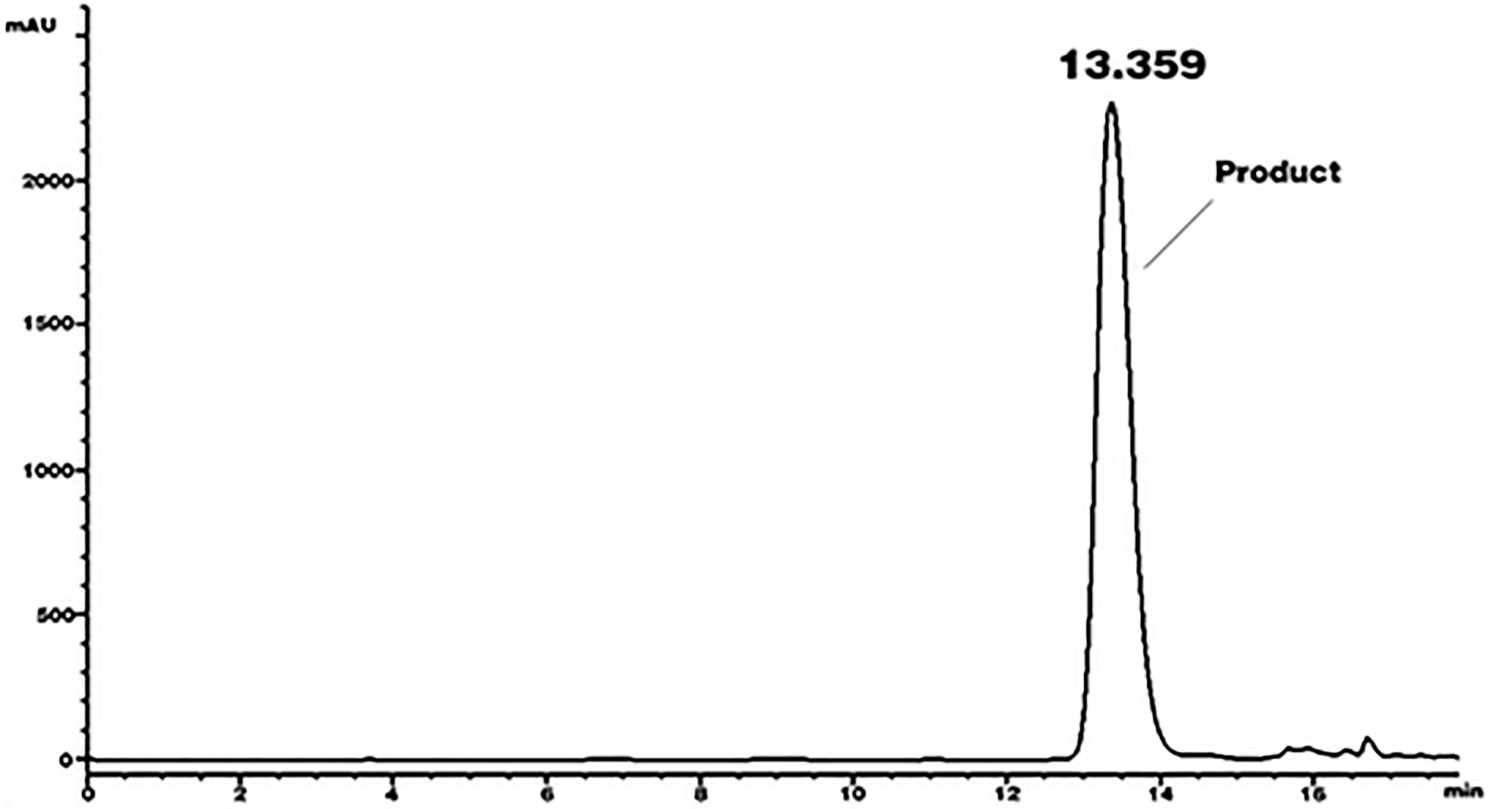
HPLC chromatogram of 5,7,8,4’-tetrahydroxyisoflavone after purification by semi-preparative HPLC.

Crude extract was treated with ethyl acetate from the bioconversion mixture. The ethyl acetate extract was then purified by using half preparative chromatograph using a 250 × 10 mm i.d., ODS 2 Spherisorb semipreparative C18 reversed-phase column.Peak area of impurity decreased obviously. And the purity of 5, 7, 8, 4’-tetrahydroxyisoflavone reached to 96% by calculation of percent of peak area (Fig 7). The elution of peak was collected by using vacuum freeze drying and 5,7,8,4′-tetrahydroxyisoflavone was identified by mass and NMR spectrometry.

## Conclusion

Our study has screened and identified a strain named *Aspergillus aculeatus* on the basis of colony morphology features and ITS rDNA sequence analysis. Phylogenetic tree was constructed to determine its taxonomic status.

Fructus sophorae known as chinese traditional medicine have a large content of isoflavone. And it is feasible to produce 5, 7, 8, 4’-tetrahydroxyisoflavone from fructus sophorae power with *Aspergillus aculeatus*. Our study describes a new methods for extraction of 5, 7, 8, 4’-tetrahydroxyisoflavone from genistein and glycosides by *Aspergillus aculeatus*. And 120 mg of 5, 7, 8, 4’-tetrahydroxyisoflavone was made from 20 g fructus sophorae powder, and the purity reached to 96%.

In the early fermentation, content of genistin of fructus sophorae decreased while the proportion of genistein increased. Then genistein decreased while 5, 7, 8, 4’-tetrahydroxyisoflavone increased with the process of fermentation. Genistein may be intermediates with *Aspergillus aculeatus* which could produce hydroxylase enzyme during fermentation.

The formation process and mechanism of isoflavones and their derivative during fructus sophorae fermentation with *Aspergillus aculeatus* should be further investigated.

## References

[1] Huamei W, Jianbo W. The Study in the Development of the Traditional Chinese Medicine Fructus Sophorae. Asia-Pacific Traditional Medicine, 2010; 6(3): 115–119.

[2] Wang ZL, Sun JY, Wang DN, Xie YH, Wang SW, Zhao WM. Pharmacological studies of the large-scaled purified genistein from Huaijiao (Sophora japonica-Leguminosae) on anti-osteoporosis. Phytomedicine. 2006; 13(9): 718–723. 17085294 10.1016/j.phymed.2005.09.005

[3] Coward L, Barnes NC, Setchell R, Barnes S. Genistein, daidzein, and their β-glycoside conjugates: Antitumor isoflavones in soybean foods from American and Asian diets. Journal of Agricultural and Food Chemistry, 1993; 41 (11): 1961–7. 10.1021/jf00035a027

[4] Kaufman PB, Duke JA, Brielmann H, Boik J, Hoyt JE. A Comparative Survey of Leguminous Plants as Sources of the Isoflavones, Genistein and Daidzein: Implications for Human Nutrition and Health. The Journal of Alternative and Complementary Medicine. 1997; 3 (1): 7–12. 10.1089/acm.1997.3.7 . 9395689

[5] Rao HSP, Reddy KS. Isoflavones from Flemingia vestita. Fitoterapia. 1991; 62 (5): 458.

[6] Rao KN, Srimannarayana G. Fleminone, a flavanone from the stems of Flemingia macrophylla. Phytochemistry. 1983; 22 (10): 2287–90. 10.1016/S0031-9422(00)80163-6

[7] Wang BS, Juang LJ, Yang JJ, Chen LY, Tai HM, Huang MH. Antioxidant and Antityrosinase Activity of Flemingia macrophylla and Glycine tomentella Roots. Evidence-Based Complementary and Alternative Medicine. 2012; 1–7. 10.1155/2012/431081 . 22997529 PMC3444970

[8] Alves RC, Almeida IMC, Casal S, Oliveira M, Beatriz PP. Isoflavones in Coffee: Influence of Species, Roast Degree, and Brewing Method. Journal of Agricultural and Food Chemistry. 2010; 58 (5): 3002–7. 10.1021/jf9039205 . 20131840

[9] Fedoreyev SA, Pokushalova TV, Veselova MV, Glebko LI, Kulesh NI, Muzarok TI, et al Isoflavonoid production by callus cultures of Maackia amurensis. Fitoterapia. 2000; 71 (4): 365–72. 10.1016/S0367-326X(00)00129-5 10925005

[10] Sanjeev B, Yuxiang Z, Shadan A, Mohammad B, Zhiwei W, Paul J, et al Molecular evidence for increased antitumor activity of gemcitabine by genistein invitro and invivo using an or thotopic model of pancreatic cancer. Cancer Research, 2005; 65(19): 9064–9072. 10.1158/0008-5472.CAN-05-1330 16204081

[11] Jingqun H, Miaozhang Z, Siwang W. Studies on Genistein about action of anti-inflammatory, analgesia and detumescence. Northwest Pharmaceutical Journal, 2011; 26(3): 193–195.

[12] Sabine EK, Doris MH, Manfred M. Oxidative metabolism of the soy isoflavones daidzein and genistein in humans in vitro and in vivo. Journal of Agriculture and Food, 2001; 49(6): 3024–3033. 10.1021/jf0012695 11410004

[13] Sabine S, Sandra R, Simone W, Ulrich M, Stefan M. Identification of a Saccharomyces cerevisiae Glucosidase That Hydrolyzes Flavonoid Glucosides. Applied and environmental microbiology, 2011; 77(5): 1751–1757. 10.1128/AEM.01125-10 21216897 PMC3067276

[14] Te-sheng C. Two potent suicide substrates of mushroom tyrosinase: 5,7,8,4-Trihydroxyisoflavone and 5,7,8,4’-Tetrahydroxyisoflavone. Journal of agricultural and food chemistry, 2007; 55(5): 2010–2015. 17295516 10.1021/jf063095i

[15] Te-sheng C. Mushroom tyrosinase inhibitory effects isofisoavone sisolated from soygerm koji fermented with *Aspergillus oryzae* BCRC32288. Journal of agricultural and food chemistry, 2007; 105:1430–1438.

[16] Vasilis PA, Ketan R, Randolph RJA, Aristidis MT, Denetrios AS. CYP1-mediated antiproliferative activity of dietary flavonoids in MDA-MB-468 breast cancer cells. Toxicology, 2009; 264: 162–170. 10.1016/j.tox.2009.07.023 19666078

[17] Yuri L, Jisun O, Yong SJ. Lactobacillus plantarum-mediated conversion of flavonoid glycosides into flavonols, quercetin, and kaempferol in cudrania tricuspidata leaves. Food Science and Biotechnology, 2015; 24(5): 1817–1821.

[18] Feng C, Shuang J, Xin XX, Yue G, Meng L, Yuan GZ, et al Effective bioconversion of sophoricoside to genistein from *Fructus sophorae* using immobilized *Aspergillus niger* and *Yeast*. World Journal of Microbiol and Biotechnol, 2015; 31: 187–197. 10.1007/s11274-014-1777-y 25392205

[19] Te-sheng C, Hsiou-Yu D, Sorgan Shou-Ku T, Ching-Yi W. Metabolism of the soy isoflavones daidzein and genistein by fungi used in the preparation of various fermented soybean foods. Bioscience Biotechnol. Biochemical, 2007; 71(5): 1330–1333. 10.1271/bbb.60573 17485838

[20] Peder B, Hans PHH, Borge D. On the safety of *Aspergillus oryzae*: a review. Applied Microbiology and Biotechnology, 1992; 36: 569–572. 1368061 10.1007/BF00183230

